# 2,5-Dimethyl­anilinium nitrate

**DOI:** 10.1107/S1600536809027718

**Published:** 2009-07-18

**Authors:** Wajda Smirani, Mohamed Rzaigui

**Affiliations:** a Laboratoire de Chimie des Matériaux, Faculté des Sciences de Bizerte, 7021 Zarzouna Bizerte, Tunisia

## Abstract

In the title salt, C_8_H_12_N^+^·NO_3_^−^, all non-H atoms of the cation lie on mirror planes. The nitrate counteranion has *m* symmetry and acts as a hydrogen-bond acceptor of N—H⋯O hydrogen bonds, connecting the cations and anions into layers running parallel to the *ab* plane.

## Related literature

Inorganic–organic hybrid materials display a great variety of structural topologies, see: Xiao *et al.* (2005 ▶). For comparative geometrical data in structures containing the same organic groups, see: Smirani & Rzaigui (2009 ▶); Souissi *et al.* (2009 ▶).
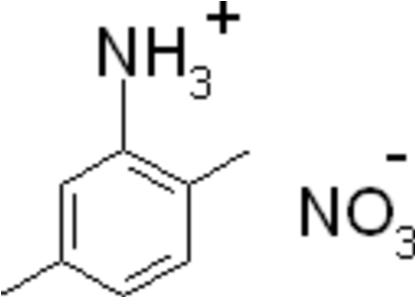


## Experimental

### 

#### Crystal data

C_8_H_12_N^+^·NO_3_^−^
*M* *_r_* = 184.20
Orthorhombic, 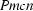

*a* = 6.762 (3) Å
*b* = 7.942 (3) Å
*c* = 17.137 (5) Å
*V* = 920.4 (6) Å^3^
*Z* = 4
Ag *K*α radiation
μ = 0.06 mm^−1^
*T* = 293 K
0.50 × 0.45 × 0.40 mm

#### Data collection

Enraf–Nonius TurboCAD-4 diffractometer
Absorption correction: none
4249 measured reflections
2365 independent reflections
822 reflections with *I* > 2σ(*I*)
*R*_int_ = 0.056
2 standard reflections frequency: 120 min intensity decay: 5%

#### Refinement

*R*[*F* ^2^ > 2σ(*F* ^2^)] = 0.054
*wR*(*F* ^2^) = 0.156
*S* = 0.92
2365 reflections
86 parameters
H-atom parameters constrained
Δρ_max_ = 0.20 e Å^−3^
Δρ_min_ = −0.21 e Å^−3^

### 

Data collection: *CAD-4 EXPRESS* (Enraf–Nonius, 1994 ▶); cell refinement: *CAD-4 EXPRESS* ; data reduction: *XCAD4* (Harms & Wocadlo, 1995 ▶); program(s) used to solve structure: *SHELXS97* (Sheldrick, 2008 ▶); program(s) used to refine structure: *SHELXL97* (Sheldrick, 2008 ▶); molecular graphics: *ORTEP-3 for Windows* (Farrugia, 1997 ▶); software used to prepare material for publication: *WinGX* (Farrugia, 1999 ▶).

## Supplementary Material

Crystal structure: contains datablocks I, global. DOI: 10.1107/S1600536809027718/hg2534sup1.cif

Structure factors: contains datablocks I. DOI: 10.1107/S1600536809027718/hg2534Isup2.hkl

Additional supplementary materials:  crystallographic information; 3D view; checkCIF report

## Figures and Tables

**Table 1:** Hydrogen-bond geometry (Å, °)

*D*—H⋯*A*	*D*—H	H⋯*A*	*D*⋯*A*	*D*—H⋯*A*
N1—H1*A*⋯O1^i^	0.95 (2)	1.92 (3)	2.870 (2)	179 (3)
N1—H2*A*⋯O1^ii^	0.89 (3)	2.24 (3)	3.037 (3)	149.7 (8)

Symmetry codes: (i) 
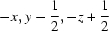
; (ii) 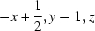.

**Fig. 1.**
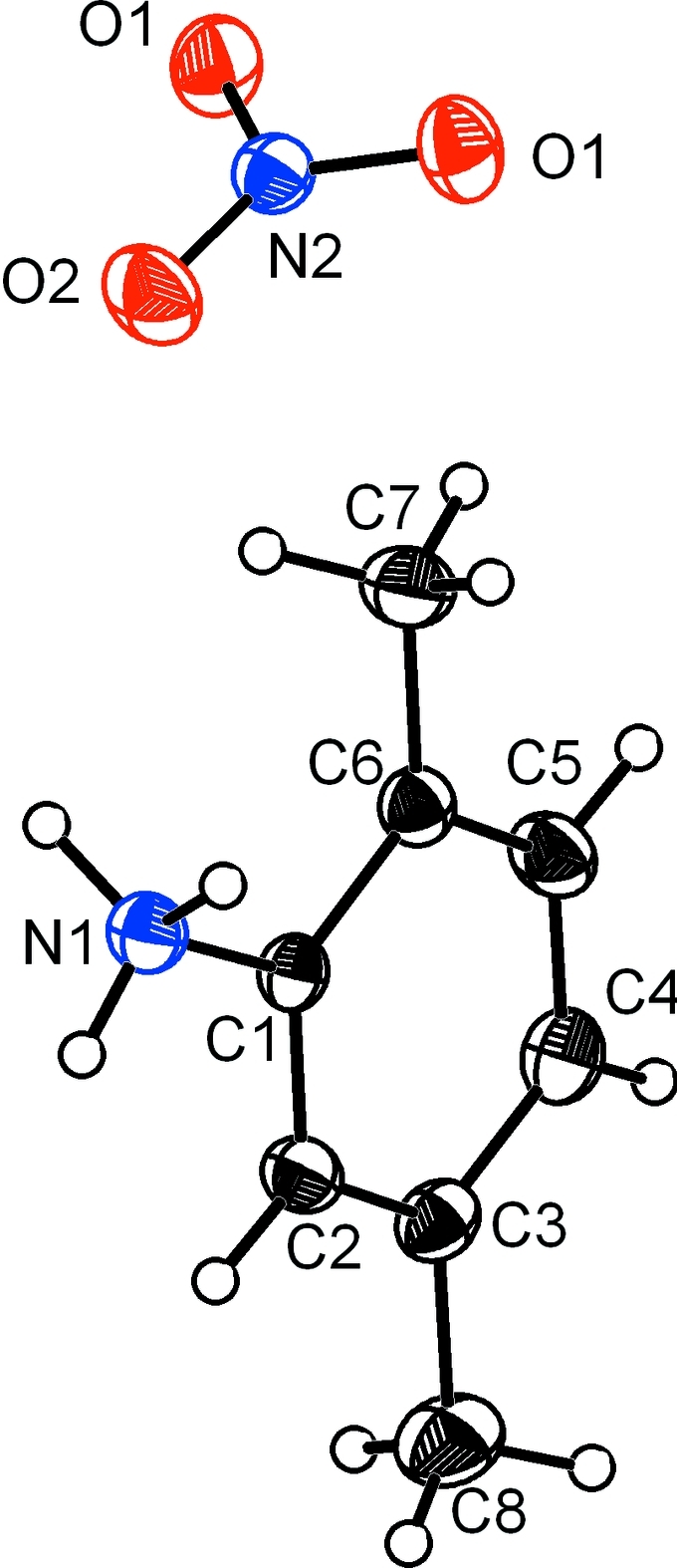
ORTEP-3 (Farrugia,(1999)) view of (C8H12N)N03 with atom numbering scheme. Displacement ellipsoids for non-H atoms are drawn at the 30% probability level.

**Fig. 2.**
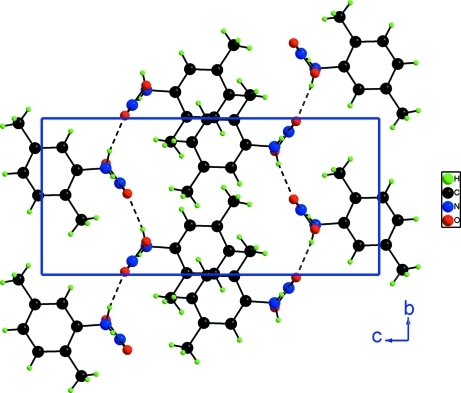
A view of the atomic arrangement of the title compound along the a axis.

## supplementary crystallographic information

### Comment

The combination of organic molecules and inorganic materials was the starting point for the developpement of new hybrid compounds with appropriate physical and chemical properties. These materials have a great interest due to their enormous variety of intriguing structural topologies (Xiao *et al.*, 2005). In order to enrich the varieties in such kinds of hybrid materials and to investigate the influence of hydrogen bonds on the structural features, we report the crystal structure of 2,5 dimethylanilinium nitrate (I).

The title compound crystallizes in the space group Pcmn. Only the non-hydrogen atoms of the cation lie on the mirror planes. As shown in Fig. 1, the asymmetric unit of the crystal structure of this salt is built of half nitrate anion and half 2,5-dimethylanilinium cation. A projection of the structure along the *a* axis shows that the nitrate anions establish with the ammonium cations multiple hydrogen bonds, to form two inorganic layers at *z* = 1/4 and 3/4.

The examination of the organic cation shows that the values of the N—C, C—C distances and N—C—C, C—C—C angles range from 1.379 (4) to 1.516 (5) Å and 116. 2(3) to 122.4 (3)°, respectively. These values are similar to those obtained in other organic materials containing the same organic groups (Smirani and Rzaigui, 2009; Souissi *et al.* 2009).

### Experimental

An ethanolic solution of 2,5-dimethylaniline (10 mmol, in 5 ml) was added drop wise to a magnetically stirred aqueous solution of nitric acid HNO_3_ (1 *M*, 10 ml) in equimolar ratio. The so-obtained solution is then filtered to eliminate the white precipitated formed and then stirred for 1 h. After stirring, the reaction mixture was kept at room temperature until apparition of transparent single crystals of 2,5-dimethylanilinium nitrate.

### Refinement

The nitrogen H atoms were located in a difference map and freely refined. The other H atoms were positioned geometrically(C–H = 0.93–0.96 Å) and refined as riding with *U*_iso_(H) = 1.2Ueq (C) or 1.5 *U*_eq_(methyl C).

### Crystal data

**Table:**

C_8_H_12_N^+^·NO_3_^−^	*F*(000) = 392
*M**_r_* = 184.20	*D*_x_ = 1.329 Mg m^−^^3^
Orthorhombic, *P**m**c**n*	Ag *K*α radiation, λ = 0.56085 Å
Hall symbol: -P 2n 2a	Cell parameters from 25 reflections
*a* = 6.762 (3) Å	θ = 9.0–10.5°
*b* = 7.942 (3) Å	µ = 0.06 mm^−^^1^
*c* = 17.137 (5) Å	*T* = 293 K
*V* = 920.4 (6) Å^3^	Block, colorless
*Z* = 4	0.50 × 0.45 × 0.40 mm

### Data collection

**Table:**

Enraf–Nonius TurboCAD-4 diffractometer	*R*_int_ = 0.056
Radiation source: fine-focus sealed tube	θ_max_ = 28.0°, θ_min_ = 2.2°
graphite	*h* = −8→11
Non–profiled ω scans	*k* = 0→13
4249 measured reflections	*l* = 0→28
2365 independent reflections	2 standard reflections every 120 min
822 reflections with *I* > 2σ(*I*)	intensity decay: 5%

### Refinement

**Table:**

Refinement on *F*^2^	Secondary atom site location: difference Fourier map
Least-squares matrix: full	Hydrogen site location: inferred from neighbouring sites
*R*[*F*^2^ > 2σ(*F*^2^)] = 0.054	H-atom parameters constrained
*wR*(*F*^2^) = 0.156	*w* = 1/[σ^2^(*F*_o_^2^) + (0.0632*P*)^2^] where *P* = (*F*_o_^2^ + 2*F*_c_^2^)/3
*S* = 0.92	(Δ/σ)_max_ < 0.001
2365 reflections	Δρ_max_ = 0.20 e Å^−^^3^
86 parameters	Δρ_min_ = −0.21 e Å^−^^3^
0 restraints	Extinction correction: *SHELXL97* (Sheldrick, 2008), Fc^*^=kFc[1+0.001xFc^2^λ^3^/sin(2θ)]^-1/4^
Primary atom site location: structure-invariant direct methods	Extinction coefficient: 0.166 (13)

### Special details

**Table:**

Geometry. All e.s.d.'s (except the e.s.d. in the dihedral angle between two l.s. planes) are estimated using the full covariance matrix. The cell e.s.d.'s are taken into account individually in the estimation of e.s.d.'s in distances, angles and torsion angles; correlations between e.s.d.'s in cell parameters are only used when they are defined by crystal symmetry. An approximate (isotropic) treatment of cell e.s.d.'s is used for estimating e.s.d.'s involving l.s. planes.
Refinement. Refinement of *F*^2^ against ALL reflections. The weighted *R*-factor *wR* and goodness of fit *S* are based on *F*^2^, conventional *R*-factors *R* are based on *F*, with *F* set to zero for negative *F*^2^. The threshold expression of *F*^2^ > σ(*F*^2^) is used only for calculating *R*-factors(gt) *etc*. and is not relevant to the choice of reflections for refinement. *R*-factors based on *F*^2^ are statistically about twice as large as those based on *F*, and *R*- factors based on ALL data will be even larger.

### Fractional atomic coordinates and isotropic or equivalent isotropic displacement parameters (Å^2^)

**Table:**

	*x*	*y*	*z*	*U*_iso_*/*U*_eq_	Occ. (<1)
H1A	0.135 (3)	0.372 (3)	0.2106 (10)	0.086 (7)*	
H2A	0.2500	0.209 (4)	0.2009 (14)	0.068 (8)*	
C6	0.2500	0.4899 (2)	0.07043 (11)	0.0405 (5)	
N1	0.2500	0.3185 (3)	0.18968 (10)	0.0427 (4)	
C1	0.2500	0.3315 (2)	0.10432 (10)	0.0349 (4)	
C2	0.2500	0.1853 (3)	0.06089 (12)	0.0432 (5)	
H2	0.2500	0.0817	0.0862	0.052*	
C5	0.2500	0.4935 (3)	−0.01074 (13)	0.0507 (6)	
H5	0.2500	0.5971	−0.0361	0.061*	
C3	0.2500	0.1907 (3)	−0.01996 (12)	0.0452 (5)	
C4	0.2500	0.3481 (3)	−0.05467 (12)	0.0499 (6)	
H4	0.2500	0.3559	−0.1088	0.060*	
C7	0.2500	0.6490 (3)	0.11749 (13)	0.0550 (6)	
H7A	0.3818	0.6722	0.1353	0.083*	0.50
H7B	0.1638	0.6361	0.1616	0.083*	0.50
H7C	0.2044	0.7406	0.0857	0.083*	0.50
C8	0.2500	0.0315 (3)	−0.06851 (15)	0.0685 (7)	
H8A	0.1164	0.0016	−0.0814	0.103*	0.50
H8B	0.3098	−0.0582	−0.0393	0.103*	0.50
H8C	0.3238	0.0501	−0.1156	0.103*	0.50
N2	0.2500	0.9146 (2)	0.26822 (9)	0.0425 (4)	
O1	0.09203 (15)	0.98204 (16)	0.24632 (7)	0.0604 (4)	
O2	0.2500	0.7900 (2)	0.30947 (10)	0.0672 (5)	

### Atomic displacement parameters (Å^2^)

**Table:**

	*U*^11^	*U*^22^	*U*^33^	*U*^12^	*U*^13^	*U*^23^
C6	0.0373 (10)	0.0419 (11)	0.0424 (10)	0.000	0.000	0.0018 (9)
N1	0.0501 (10)	0.0419 (11)	0.0360 (9)	0.000	0.000	0.0017 (8)
C1	0.0330 (9)	0.0399 (10)	0.0318 (9)	0.000	0.000	0.0012 (8)
C2	0.0458 (11)	0.0363 (11)	0.0476 (11)	0.000	0.000	0.0020 (9)
C5	0.0620 (15)	0.0427 (11)	0.0474 (12)	0.000	0.000	0.0089 (10)
C3	0.0411 (11)	0.0505 (13)	0.0440 (11)	0.000	0.000	−0.0092 (10)
C4	0.0506 (12)	0.0630 (15)	0.0361 (10)	0.000	0.000	0.0019 (10)
C7	0.0642 (14)	0.0425 (12)	0.0584 (13)	0.000	0.000	−0.0034 (11)
C8	0.0796 (19)	0.0673 (16)	0.0586 (14)	0.000	0.000	−0.0229 (13)
N2	0.0476 (10)	0.0425 (10)	0.0374 (9)	0.000	0.000	−0.0025 (8)
O1	0.0435 (6)	0.0667 (9)	0.0708 (7)	0.0079 (5)	0.0007 (6)	0.0138 (6)
O2	0.0868 (13)	0.0550 (10)	0.0598 (10)	0.000	0.000	0.0189 (9)

### Geometric parameters (Å, °)

**Table:**

C6—C1	1.385 (3)	C3—C8	1.514 (3)
C6—C5	1.391 (3)	C4—H4	0.9300
C6—C7	1.499 (3)	C7—H7A	0.9600
N1—C1	1.467 (2)	C7—H7B	0.9600
N1—H1A	0.95 (2)	C7—H7C	0.9600
N1—H2A	0.89 (3)	C8—H8A	0.9600
C1—C2	1.379 (3)	C8—H8B	0.9600
C2—C3	1.386 (3)	C8—H8C	0.9600
C2—H2	0.9300	N2—O2	1.216 (2)
C5—C4	1.379 (3)	N2—O1	1.2525 (14)
C5—H5	0.9300	N2—O1^i^	1.2525 (14)
C3—C4	1.384 (3)		
			
C1—C6—C5	115.98 (18)	C5—C4—C3	121.46 (19)
C1—C6—C7	122.67 (18)	C5—C4—H4	119.3
C5—C6—C7	121.35 (19)	C3—C4—H4	119.3
C1—N1—H1A	110.2 (11)	C6—C7—H7A	109.5
C1—N1—H2A	106.6 (16)	C6—C7—H7B	109.5
H1A—N1—H2A	110.6 (14)	H7A—C7—H7B	109.5
C2—C1—C6	122.56 (17)	C6—C7—H7C	109.5
C2—C1—N1	118.60 (18)	H7A—C7—H7C	109.5
C6—C1—N1	118.84 (17)	H7B—C7—H7C	109.5
C1—C2—C3	120.9 (2)	C3—C8—H8A	109.5
C1—C2—H2	119.6	C3—C8—H8B	109.5
C3—C2—H2	119.6	H8A—C8—H8B	109.5
C4—C5—C6	121.9 (2)	C3—C8—H8C	109.5
C4—C5—H5	119.1	H8A—C8—H8C	109.5
C6—C5—H5	119.1	H8B—C8—H8C	109.5
C4—C3—C2	117.2 (2)	O2—N2—O1	121.48 (9)
C4—C3—C8	121.2 (2)	O2—N2—O1^i^	121.48 (9)
C2—C3—C8	121.6 (2)	O1—N2—O1^i^	117.04 (17)
			
C5—C6—C1—C2	0.0	C7—C6—C5—C4	180.0
C7—C6—C1—C2	180.0	C1—C2—C3—C4	0.0
C5—C6—C1—N1	180.0	C1—C2—C3—C8	180.0
C7—C6—C1—N1	0.0	C6—C5—C4—C3	0.0
C6—C1—C2—C3	0.0	C2—C3—C4—C5	0.0
N1—C1—C2—C3	180.0	C8—C3—C4—C5	180.0
C1—C6—C5—C4	0.0		

Symmetry codes: (i) −*x*+1/2, *y*, *z*.

### Hydrogen-bond geometry (Å, °)

**Table:**

*D*—H···*A*	*D*—H	H···*A*	*D*···*A*	*D*—H···*A*
N1—H1A···O1^ii^	0.95 (2)	1.92 (3)	2.870 (2)	179 (3)
N1—H2A···O1^iii^	0.89 (3)	2.24 (3)	3.037 (3)	150 (1)

Symmetry codes: (ii) −*x*, *y*−1/2, −*z*+1/2; (iii) −*x*+1/2, *y*−1, *z*.
